# Effect of Two Different Rehabilitation Approaches on Pulmonary Functional Tests, Neuromuscular Functions and Quality of Life in Juvenile Myasthenia Gravis: A Randomized Controlled Trial Study

**DOI:** 10.3390/medicina58030374

**Published:** 2022-03-02

**Authors:** Rasha A. Mohamed, El Sayed H. Mohamed, Mohamed A. Basiouny, Ibrahim M. Hamoda, El Sayed M. Hanoura, Elbadawy I. Elhenedi, Abd El Aziz A. Sherief

**Affiliations:** 1 Department of Physical Therapy for Growth and Developmental Disorders in Children and Its Surgery, Faculty of Physical Therapy, Cairo University, Cairo 12613, Egypt; 2 Department of Physical Therapy, Cardiopulmonary Disorders, Buraydah Private Colleges, Buraydah 51418, Saudi Arabia; dr.elsayedhassanpt@gmail.com; 3 Department of Neurology and Neuropsychiatry, Faculty of Medicine, Tanta University, Tanta 31527, Egypt; mohamed.bassiouny@med.tanta.eg; 4 Department of Physical Therapy for Neuromuscular Disorders and Its Surgery, Faculty of Physical Therapy, Kafr Al Sheikh University, Kafrelsheikh 33516, Egypt; shiny_str@yahoo.com; 5 Department of Physical Therapy for Internal Medicine, Faculty of Physical Therapy, Kafr Al Sheikh University, Kafrelsheikh 33516, Egypt; dr.sayedhanora@gmail.com; 6 Department of Physical Therapy for Neuromuscular Disorders and Its Surgery, Faculty of Physical Therapy, Cairo University, Cairo 12613, Egypt; jeham2013@gmail.com; 7 Department of Physical Therapy for Growth and Developmental Disorders in Children and Its Surgery, Faculty of Physical Therapy, Kafr Al Sheikh University, Kafrelsheikh 33516, Egypt; aabdelazez10@yahoo.com

**Keywords:** action potentials, fatigue, muscle weakness, myasthenia gravis, quality of life, respiratory insufficiency

## Abstract

*Background and Objectives*: Children with juvenile myasthenia gravis have a variety of symptoms, ranging from isolated intermittent ocular complaints to overall muscle weakness with or without respiratory insufficiency. This study aimed to investigate the efficacy of a specialized physical therapy with or without partial body weight supported treadmill training on pulmonary functional tests, neuromuscular functions, and quality of life. *Materials and Methods*: Thirty children, ranging in age from 13 to 16 years, were distributed randomly into two study groups (A or B). Both groups underwent a designed physical therapy program. In addition, group A underwent the partial body weight supported treadmill training. The treatment was conducted three times a week for 12 weeks successively. Pulmonary functional tests (FVC, FEV1, PEFR, and MVV), neuromuscular function tests (compound motor action potential, isometric muscle force of biceps brachii and rectus femoris, balance, walking endurance, and fatigue), and quality of life were measured before and after 12 successive weeks. *Results*: A significant improvement in all investigated variables were recorded in both groups in favor of group A. *Conclusions*: Both a specialized physical therapy and partial body weight supported treadmill training are effective in terms of enhancing pulmonary functional tests, neuromuscular functions, and quality of life. Partial body weight supported treadmill training is an excellent adjunctive to the physical therapy program.

## 1. Introduction

One of the uncommon autoimmune-related neuromuscular disorders is myasthenia gravis (MG). It is characterized by the presence of antibodies directed against the cholinergic receptor [1]. Patients have a variety of symptoms, ranging from isolated intermittent ocular complaints to overall muscle weakness with or without respiratory insufficiency [2]. Juvenile myasthenia gravis (JMG) is subdivided into pre-pubertal (before the age of 12 years) and post-pubertal (after the age of 12 years), based on the patient’s first occurrence of symptoms. [3]. Skeletal muscle weakness increases with fatigue throughout the day. It can be localized or generalized, and it is frequently more proximal than distal [4]. These symptoms have varied degrees of impact on patients [5]. Fatigue correlated to a deterioration in disease, a higher risk of depression, and lower health-related quality of life (HRQoL). It may set off a vicious cycle by lowering physical activity, which has negative consequences for muscular strength and fatigue [6]. 

MG patients frequently exhibit a “myasthenic pattern,” in which respiratory volumes and total lung capacity decrease during a rapid shallow breathing pattern at rest or maximal voluntary ventilation (MVV) [7]. Dyspnea and poor exercise tolerance are caused by respiratory muscle weakness and fatigue, which can impact HRQoL [8]. 

In comparison to healthy people, patients with MG have a more sedentary lifestyle. This, among other things, affects bone density—resulting in falls and fractures [1]. The ability to maintain balance is a multifaceted motor skill. The integration of sensory inputs and their processing in the central nervous system is the basis for balance control [9]. The sensory system is composed of several different muscle, joint, and cutaneous mechanoreceptors [10]. The strength necessary for the movement of the extremities is generated, collected, and transferred to the upper extremity from the lower extremity by the postural control [11]. The somatosensory input disorders can disrupt the postural control and lead to falls in neuromuscular diseases, resulting in proprioceptive insufficiency [10].

People with MG, particularly those with mild-to-moderate degrees, should be offered a comprehensive rehabilitation approach to improve functional independence, reduce secondary medical comorbidities, avoid or restrict deformities, and help the patient integrate into the society [12,13,14]. 

Respiratory muscle training (RMT) might help to enhance the endurance and strength of the respiratory muscles. It can be used safely and efficiently with mild-to-moderate MG patients (classes II and III) [15,16]. Balance training is an essential aspect of the MG rehabilitation process [1]. Exercise treatment aims to enhance functional outcomes (muscle strength, mobility, and aerobic capacity), fatigue, physical performance, and HRQoL in patients with MG [17]. Patients with mild generalized adult MG could tolerate aerobic or strengthening exercises [18]. 

Treadmill training has advantages in that exercise loads could be adjusted by changing rotation speeds or slopes, allowing accurate calculation of exercise loads for repeated measurement, engaging a large number of muscles, and improving physical capacities through cardiopulmonary function exercise [19,20]. The use of an overhead harness to carry a part of body weight during walking on a treadmill is known as partial body weight supported treadmill training (PBWSTT). It focuses on gait training for strength, endurance, and task-specific tasks. Task-oriented training allows multiple repetitions of supervised walking with virtually no risk of falling, improving postural control and cardiopulmonary capacity [21].

A thorough meta-analysis of the scientific literature showed that a combination of aerobic and strengthening exercises are effective for patients with different primary muscle diseases [22]. Several previous studies on children with different disorders found that PBWSTT could result in improving the postural control, some gait parameters, and activity/participation without any negative side effects [23,24,25,26]. 

To our knowledge, there is a lack of studies to investigate the impact of exercises on JMG. Therefore, the aim of this study was to investigate the efficacy of a specialized physical therapy program with or without PBWSTT on pulmonary functional tests (PFTs), neuromuscular functions, and quality of life (QoL) in children with JMG.

The primary hypothesis was that either a specialized physical exercise program or PBWSTT would improve PFTs, neuromuscular functions, and QoL of children with JMG. The secondary hypothesis was that PBWSTT in conjunction with a specialized physical therapy program would be more effective than a specialized physical exercise program alone.

## 2. Materials and Methods

### 2.1. Study Design

This is a controlled, randomized, and parallel group study. It was conducted for 12 weeks successively in the Outpatient Clinic of El-Shatby University Hospital, Alexandria and Faculty of Physical Therapy, Kafr Al Sheikh University, Egypt. The study was reported according to the guidelines of the Consolidated Standards of Reporting Trials (CONSORT) and the Standard Protocol Items: Recommendations for Interventional Trials (SPIRIT) [27,28]. 

### 2.2. Participants

Thirty patients were recruited from Outpatient Clinic of El-Shatby University Hospital, Alexandria. Their ages ranged from 13 to 16 years (5 boys, 25 girls) and were recruited consecutively. This post-pubertal type of JMG typically presents in adolescent girls [29]. Eligibility for participation include the following: mild-to-moderate JMG according to The Myasthenia Gravis Foundation of America clinical classification [30]. They were diagnosed based on clinical examination, serological criteria, and neurophysiological studies. They had generalized muscle weakness and positive anti-nicotinic acetylcholine receptor (anti-nAchR) antibodies. MG was confirmed by repetitive nerve stimulation (RNS). Their symptoms started after 12 years of age and were stable for at least six months before the beginning of the study, measured using Quantitative Myasthenia Gravis Score (QMG). All the patients were on pyridostigmine medication.

The exclusion criteria included: chronic pain, severe cardiovascular disease, thymectomy, hospitalized patients, severe orthopedic conditions, respiratory diseases, hospitalization in the preceding three months for a medical or surgical condition, Graves’ disease, anemia (hematocrit < 30%), antibody positive thyroid disease, thymus hyperplasia, diplopia, participation in another interventional clinical study in the last three months, or difficulty to deal with the tests and intervention protocol after the familiarity sessions.

### 2.3. Sample Size Calculation

G*POWER statistical software (Version 3.1.9.2; Franz Faul, Universitat Kiel, Germany) was used to calculate the sample size. Based on the data of isometric muscle force from a pilot study which was conducted on 5 subjects in each group, it revealed that the sample size required for this study was 15 patients for each group. α = 0.05, β = 0.2, effect size = 1.1, and allocation ratio N2/N1 = 1 were used calculations.

### 2.4. Randomisation, Allocation and Blinding

**Figure 1:**
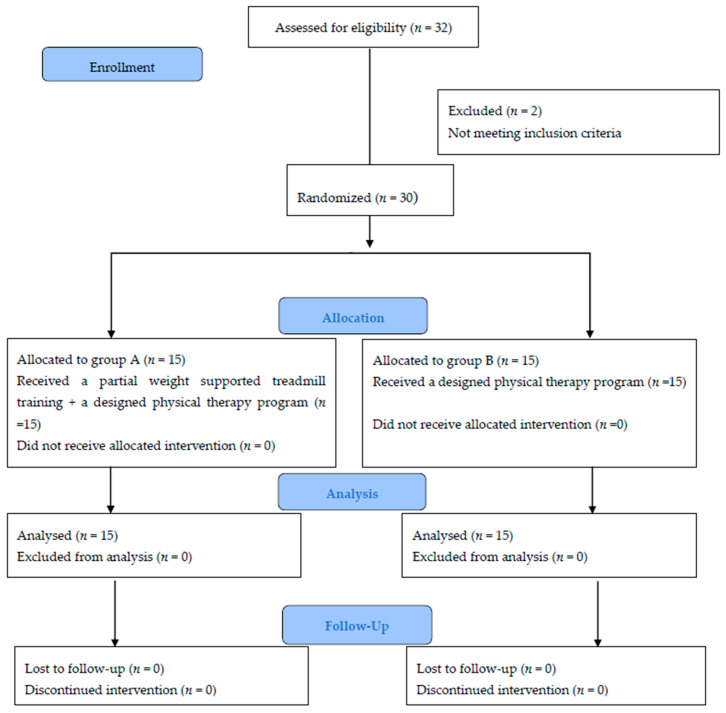
Flow chart of the study design.

The flow chart of patients through the trial, assessments, and interventions timeline are displayed in Figure 1 using the CONSORT guidelines [27]. A total of 32 patients were screened for eligibility by a research coordinator. Two patients were ruled out because they did not match the eligibility requirements. All data were coded to ensure anonymity. With a 1:1 allocation ratio, thirty eligible patients were randomly allocated to either group A or B. A computer-generated randomization scheme was utilized, with randomly permuted blocks stratified by the center. The block sizes were not revealed in order to maintain confidentiality. The non-blinded physiotherapist, who was not involved in the study, was given the group allocation using computer software (CleanWeb). The physiotherapist informed the patient verbally after the randomization (concealed allocation) was completed. An off-site independent statistician, who was not involved clinically in the trial, created the randomization list prior to the start of the study. 

Blinded assessors conducted clinical and neurophysiological evaluations. The author who was responsible about the measurements was blinded of the group assignment for clinical evaluations. Patients were told not to tell their neurologist or assessing author about their assignment. All patients were asked to keep their regular lifestyle throughout the research. After allocation, no children dropped out of the study. 

### 2.5. Outcome Measures

Candidates for the study were first assessed through a medical history and physical examination. Height (cm) and weight (kg) were measured using health scale 70, made in China. Then, a calculation of the body mass index (BMI) was performed.

Assessments were conducted for all the participating patients in both groups before and after 12 successive weeks of treatment. PFTs and neuromuscular functions (neurophysiological measurements, isometric muscle force, balance, walking endurance, fatigue, and HRQoL) were collected by the same author. When the patient was taking anticholinesterase medication, assessments were undertaken approximately 3 h after ingestion. Because anticholinesterase inhibitors might affect muscle strength and fatigue for a short period of time, the time of medication and evaluation was recorded. The same timing was adhered as possible to pre and post-treatment measurements. Familiarity sessions were held to show the patients how to perform the tests appropriately.

#### 2.5.1. Primary Outcome Measures

##### Pulmonary Functional Tests

Master Screen Paed Spirometery was used to perform the PFTs. Forced vital capacity (FVC), forced expiratory volume in one second (FEV1), peak expiratory flow rate (PEFR), and maximum voluntary ventilation (MVV) were all included. The patient was sitting in an upright position with a backrest, 90° flexion of the hip and knee joints, and feet were firmly planted on the floor. The same spirometer was utilized for all examinations. After each maneuver, the patient was given five minutes to relax. Each maneuver was performed three times, obtaining the highest value [31].

##### Neurophysiological Measurements

Compound motor action potential (CMAP) is an objective measure of neuromuscular function of the motor units [32]. The CMAP was recommended for JMG [33]. The proximal muscles had higher CMAP amplitudes in regularly trained individuals than in those who are untrained [32,34]. A certified neurophysiological technologist performed all CMAP. Four small circular silver–silver chloride surface electrodes with 4 mm diameter and an inter-electrode spacing of 11 mm were applied. These recording electrodes were placed in the middle of the muscular belly, parallel to the muscle fibers’ direction. The right biceps brachii (musculocutaneous nerve) and rectus femoris (femoral nerve) muscles were evaluated in their dominant side extremities. Because these muscles are primarily used in regular training regimens, they could be used to follow the muscular changes after training. For the biceps brachii, neutral reference electrodes were put on the ulnar styloid process, and, for the rectus femoris, on the fibular head or patella. Supra-maximal electrical impulses (0.2–0.3 ms) were supplied using stimulators inserted on the femoral nerve below the inguinal ligament and in the axilla (posterior border of the short head of biceps within 1 inch of axillary fold) for stimulation of the musculocutaneous nerve.

##### Isometric Muscle Force

A hand-held dynamometer was used to measure isometric muscle force (HHD, model 01165; Lafayette Instrument Company, Lafayette, IN, USA). Measuring of the peak force in kg was performed during a 5 s interval. The greatest force of three consecutive repetitions was recorded. For measuring the elbow flexion (biceps brachii), the patient laid in a supine position with the shoulder adducted, elbow flexed 90° and forearm supinated, and the dynamometer was placed on the volar side of the forearm. For measuring knee extension (quadriceps muscles), the patient was sitting with the knee joint flexed 90° and the dynamometer was situated on the anterior surface of the lower part of the tibia [32].

#### 2.5.2. Secondary Outcome Measures

##### Balance Assessment 

The Biodex stability system (BSS; Shirley, NY, USA: Biodex, Inc.) was used to detect balance problems. The BSS measured the anterior–posterior stability index (APSI), medial–lateral stability index (MLSI), and overall stability index (OSI). The BSS featured eight levels of stability, with level 1 being the least stable and level 8 being the most stable. Because the patients had problems maintaining their equilibrium below this level throughout familiarity sessions, the stability indices were evaluated at level 8. The balance measurement test was conducted three times, with the data being analyzed using the average.

##### Walking Endurance

Walking endurance was assessed using the six-minute walk test (6MWT). In several populations, including patients with neuromuscular disorders, it has been verified as a meaningful measure of functional capacity and endurance [35]. It had a good test–retest reliability [36]. It was administered in accordance with the American Thoracic Society’s (ATS) guidelines and recommendations [37]. The patients were informed to wear comfortable clothes with proper walking shoes. The patients walked 30 m for 6 min on an unobstructed and rectangular pathway at a maximum speed. They were instructed to maintain a steady pace without running. Using track markings, the six-minute walk distance (6MWD) was determined to the nearest meter. The patient was holding a pulse oximeter in his/her hand to measure Spo_2_. During the test, the timer continued to run even when there was a pause. 

##### Fatigue and Health-Related Quality of Life

Fatigue was assessed using the multidimensional fatigue scale (MFS) of the pediatric quality of life inventory (PedsQL TM) [38]. The PedsQL TM MFS is the most widely used tool for assessing fatigue in children with chronic illnesses [39]. It was available in a child self-report version and a parent proxy-report version. In this study, the self-report version was used. It is an 18-item questionnaire with three domains with 6 items for each: general fatigue, sleep/rest fatigue, and cognitive fatigue. The total number of items answered across all domains was divided by the total number of items answered to get the total score. If more than 50% of items in a domain were missing, the domain score was not calculated. Higher scores suggested that there were fewer issues or symptoms.

The Pediatric Quality of Life Inventory (PedsQL TM 4.0) was used to assess HRQoL. It was intended to assess the WHO’s core dimensions of health (physical, emotional, social, and school functioning). It is a complete and reliable measure of HRQoL in children [39]. It was previously used to assess patient-perceived well-being in boys with Duchene muscular dystrophy [40]. A total HRQoL composite score was also provided. The scores range from 0 (lowest HRQoL) to 100 (highest HRQoL).

### 2.6. Interventions

Both groups underwent the same designed physical therapy program for 1 h. In addition, group A underwent PBWSTT 30 min. The interventions were conducted for 3 times (day after day)/week for 12 successive weeks. Each intervention was performed by the same author for all patients throughout the treatment period. 

Several rest intervals were taken by the patient. When the patient complained of pain or exhaustion, the exercise was stopped, and a 2 to 3 min rest was given, which was not counted against the exercise duration. The patient was wearing a Garmin^®^ HR monitor. During the exercises, the HR should not exceed more than 30 beats over its resting level. After the exercise, the patient should not be fatigued for more than 2 h and there should be no acute muscle discomfort in the same day or the day following the exercise. If the patient was taking pyridostigmine, exercise began 1.5 to 2 h after the last dose. During the period of training, the patients’ daily activities, the medication type, and dose were not modified.

During the treatment sessions, the same author monitored and verified compliance. Before beginning the interventions, familiarity sessions were held to show the patients how to perform the exercises appropriately.

Phone calls to the patients were conducted to provide encouragement and enhance the compliance. The adherence rate depended on the attendance and completeness of the exercises. The attendance of each patient was calculated by the number of the prescribed visits attended. The completeness of the exercises was calculated by the percentage of the exercises completed to the author’s satisfaction. The template for the intervention description and replication (TIDieR) checklist [41] was used to assess adequate exercise performance during the therapy sessions. When a patient missed more than two sessions and did not complete >90% of the tasks, he or she was designated a dropout from the study. All the patients were cooperative during the study and no patients dropped out. The adherence rate was approximately 98%.

#### 2.6.1. Criteria for Discontinuation

If a patient’s MG worsened, or if there was any other medical condition or new incident for which the exercise was contraindicated, it was recommended that the patient should discontinue training. Patients were also asked to fill out a training diary in which they could record their medicine and any mild side effects from treatment sessions (such as any MG symptoms or fatigue). Patients were advised to contact their general practitioner if their symptoms increased, as well as informing the study’s author. The participating patients had no MG exacerbations during the 12-week therapy period.

#### 2.6.2. A Specialized Exercise Program

Both groups underwent the same designed physical therapy program which included the following:

##### Breathing Exercises

The patients performed diaphragmatic breathing (DB) and pursed lips breathing (PLB). The duration of breathing exercises was 25 min consisting of DB for 10 min, 5 min rest, and 10 min, and finally PLB for 5 min.

##### Physical Exercises

The same group of exercises that aimed to improve the strength, flexibility, and balance were performed for 35 min. It included the following exercises:

- Resistance exercises: latissimus dorsi pull down, biceps curl, triceps pushdown, leg curl, and leg extension were carried out. Each exercise was performed for 2 sets for 10 repetitions maximum. Individual adjustment of weight was conducted for each patient.
- Sit-to-stand while holding a small Swiss ball, stepping up and down, stepping up sideways and down, throwing and catching ball, heel-toe walking, stand on one foot (goal = 30 s), and tandem stance exercise. Each exercise was repeated 10 times.
- Stretching of hamstring, calf muscles, lower back, piriformis, pectoralis major (held for 20 s, relation for 20 s, and repeated 3 times).

#### 2.6.3. Partial Body Weight Supported Treadmill Training

The treadmill training sessions were conducted on a motorized treadmill (Proteus MTM 4500a). Each patient was strapped into a modified parachute harness with a series of straps and fittings, used to secure him or her to the PBWSTT equipment. The ceiling hoist was hooked to the harness (Bravo hoisted). The patient’s lower trunk was tightly wrapped by the support vest, permitting hip flexion and extension. 

The body weight support was gradually reduced as treatment progressed, but the speed was gradually increased. The two parameters were not altered at the same time. During training sessions, the level of body weight support (BWS) ranged from 30% to 0% of the body weight. Patients began the treatment with the device alleviating 30% of their body weight; PBWSTT greater than 30% is not suggested because it induces significant muscle activity reduction in the lower limbs. In addition, BWS by the device was gradually reduced until the patient was able to walk while attached to the support and without any relieving of body weight. The amount of BWS was calculated depending on the alignment of the trunk and limbs, proper bearing, and weight shift onto the lower limbs during the walking on the treadmill. The speed was adjusted to fit the patients’ comfort level on the treadmill. The maximum speed was gradually increased over the course of the 12 weeks, depending on the patient’s ability and capacity.

The patients’ progression was sustained as long as they could keep their trunk and limbs aligned with proper weight shift, if not, the changed parameter was returned to its previous value. 

A mirror was placed in front of the treadmill to provide feedback on postural alignment and to help motivate the patient. Initially, patients were allowed to use the treadmill’s handrail, but after that, all patients were encouraged to walk independently without support other than the BWS system. The patients were instructed to walk on the treadmill in an upright position with their head up and looking forward. 

The training session lasted for 30 min. The treatment began with a 10 min cardiovascular system warm-up on the treadmill at 0.8 miles per hour (mph) which was the suitable speed for all patients during the familiarity session. Then, the speed increased until reaching the maximum at the individual target HR (i.e., 70% of the maximal HR, HR max = 208 − 0.7 × age), with more speed. At this maximal speed, the patient walked for 15 min of constant aerobic activity (an endurance plateau phase). Finally, when cooling down for 5 min, the speed was gradually declined to zero to allow HR and breathing to return to their normal levels. 

### 2.7. Data Analysis

A between-groups comparison of the patients’ characteristics was conducted using an unpaired *t*-test. Chi squared test was conducted for comparison of sex and severity distribution. The Shapiro–Wilk test was used to check the normal distribution of data. The homogeneity of variances between groups was conducted using the Levene’s test. A mixed-model MANOVA was performed to compare within and between groups. Post hoc tests using the Bonferroni correction were carried out for subsequent multiple comparisons. The level of significance was set at *p* < 0.05 for all statistical tests. Statistical package for social studies (SPSS) Version 25 for Windows (IBM SPSS, Chicago, IL, USA) was conducted through all statistical analyses.

## 3. Results

### 3.1. Participants’ Characteristics 

There were no significant differences in patients’ age, weight, height, BMI, sex, and severity distribution between groups (*p* > 0.05) (Table 1).

### 3.2. Effect of Treatment on Both Groups

There was a significant interaction of treatment and time (F = 45.34, *p* = 0.001, $\eta^{2}$ = 0.97). Significant main effects of time (F = 961.11, *p* = 0.001, $\eta^{2}$ = 0.99) and treatment (F = 62.65, *p* = 0.001, $\eta^{2}$ = 0.98) were found. 

#### 3.2.1. Within-Group Comparison

There was a significant increase in PFTs (FVC, FEV1, PEFR, and MVV), CMAP amplitude and isometric muscle force of biceps brachii and rectus femoris, 6MWD, PedsQL TM MFS, and PedsQL of both groups post-treatment compared with pre-treatment values (*p* < 0.001) (Table 2 and Table 3). Additionally, there was a significant decrease in APSI, MLSI, and OASI of both groups (*p* < 0.001) (Table 4).

#### 3.2.2. Between-Group Comparisons

There was no significant difference between groups pre-treatment (*p* > 0.05). There were significant differences between both groups post-treatment in all measuring variables in favor of the group A (*p* < 0.001) except in CMAP amplitude of biceps brachii (*p* > 0.05).

## 4. Discussion

Respiratory and proximal skeletal muscular weakness along with fatigability may limit exercise capacity in MG [42]. The aim of this study was to investigate the efficacy of a specialized physical therapy with or without PBWSTT on PFTs, neuromuscular functions, and QoL in children with JMG.

The results showed that the intervention was reasonable and well tolerated by the patients. There was a significant improvement in all measuring variables including PFTs (FVC, FEV1, PEFR, and MVV) and neuromuscular function tests (CMAP, isometric muscle force of biceps brachii and rectus femoris, balance, 6MWT, fatigue, and HRQoL) after treatment in both groups (designed physical therapy alone or with the gait/conditioning training using PBWSTT) in favor of the group that received PBWSTT. Therefore, the results support the hypothesis of the study.

One theory for the observed improvement is that the exercises resulted in different physiological benefits such as increased skeletal muscle mass, mitochondrial amount, and enhanced lactate degradation [43,44]. Strength training causes changes in different types of muscle fibers, as well as an increase in the oxidation of fast twitch muscle fibers [45]. It also triggers an immunological response, resulting in decreasing immunoglobulin secretion, increasing T regulatory cells, and changing in the Th1/Th2 balance away from Th1 cell production. Furthermore, physical activity increases the production of the myokine (cytokine generated by skeletal muscle) IL-6, which induces an anti-inflammatory response via IL10 secretion and suppression of IL-1 [46]. These effects lead to increased neuromuscular transmission efficiency, endurance, strength, and functional activities [43,44,47]. Furthermore, patients with MS benefit from improved mood, less fatigue, and good impacts on cognition, mobility, and QoL [46]. Muscular adaptations such as enhanced protein synthesis and muscle fiber hypertrophy take place 6-8 weeks to emerge in response to strength training. Neural adaptations such as enhanced synchronization and activation of motor units begin as early as two weeks and take into account early strength gains [48].

The superior improvement in group A could be related to a combined effect of a designed exercise program and PBWSTT. In a prospective study [49], 10-week aerobic and resistance training was implemented in 10 patients with MG. Muscle strength, muscle enzymes, disease-specific enzymes, and physical fitness were improved after the training period. PBWSTT is superior to conventional therapy because it allows a greater number of steps performed within a fixed period of time, resulting in more task-specific practice [50]. PBBWS worked on a multi-sensory system basis: visual, vestibular, and proprioceptive inputs. It helps in muscle architecture changes such as increased mitochondrial number and density, skeletal muscle mass and capillary bed size, and improved lactate degradation, all of which contribute to the muscle’s force generation potential [43,51]. Walking enhanced body image, mood, and overall well-being [43]. 

The effect of 12-weeks RMT on adults with mild-to-moderate generalized MG was studied by Hsu et al. [52]. They found that PFTs (FVC, FEV1), 6MWT were improved with a reduction in fatigue. The respiratory muscles are skeletal muscles both morphologically and functionally. They may respond to training in the same way as other skeletal muscles. Muscle strength, endurance, and functional exercise capacity were all improved, resulting in a decrease in the occurrence of various MG problems, such as dyspnea, better QoL and independence in daily living activities [53,54]. The physiological effects of RMT include hypertrophy of the diaphragm, increasing the percentage of type I fibers and the size of type II fibers in the external intercostal muscles, attenuation of the respiratory muscle metaboreflex, improving neural control of the respiratory muscles [55], enhancing the capillary blood flow and oxidative capacity [56], and reducing the effort to breathe [57]. PLB alters the pattern of respiratory muscle activation, increasing engagement of chest wall accessory muscles and abdominal muscular activity throughout the breathing cycle while decreasing diaphragmatic muscle recruitment. As a result, the diaphragm’s propensity for becoming fatigued during physical activity reduces [58].

Park and Hang [59] investigated whether 12 weeks of treadmill aerobic exercise was useful in improving PFTs. Following exercise, FVC and FEV1 were significantly improved. Elnozhe et al. [60] studied the effects of treadmill-walking training combined with deep breathing exercises on pulmonary functioning in Parkinson’s patients. FEV1, FVC, and FEV1/FVC improved significantly. Because of the long-term activity of muscles during treadmill training, a large amount of oxygen consumption is required. The treadmill could increase the lung volumes through widening of the respiratory tracts and decreasing of air flow resistance, stimulation of inactive alveoli, increasing of alveolar compliance, expanding the rib cage, and strengthening of the respiratory muscles, resulting in a greater lung elasticity and lung expansion [59,61]. 

The improvement in CMAP and isometric muscular force of the proximal arm and leg muscles is consistent with prior studies of MG [18,49]. This also matches the muscular response observed in healthy, well-trained individuals [32]. A previous study found a difference in arm and leg muscle improvement [62]. The discrepancy could be explained by a higher percentage of lower limb muscle exercise. It could assume that because the quadriceps muscle is more active in daily activities, a larger degree of upper limb inactivity would necessitate a longer training session to achieve equal outcomes. Because there is a CMAP arm–leg difference related to different genders, the trained healthy females have greater CMAP amplitudes of the quadriceps while the trained healthy males have greater CMAP amplitudes of the biceps brachii in comparison to untrained people of the same gender [32]. The majority of the patients in this study were female, as well as in a previous study with the same results [17]; this could explain the arm–leg difference in response to training.

Balance training consists of activities that target the sensorimotor system in order to improve function. The results of this study reflected improvements in strength and fatigue resistance, which were linked to greater balance control. This is in line with the results of Wong et al. [63]. 

The other important finding in this study was the reduction in fatigue after treatment. There are many causes of fatigue, including in physical, emotional, and mental domains. Ventilatory dysfunction was reported as a cause of reduced exercise tolerance [64]. Therefore, increasing strength, balance, and ventilatory functions through appropriate exercises could reduce fatigue [43]. This result is in line with the findings of Alekseeva et al. [65]. In addition PBWSTT could increase the motivation and self-confidence [66].

Muscle weakness and fatigue significantly impacted on QoL [43]. The findings of this study reflect an improvement in muscle strength, fatigue, and PFTs, which could explain the improvement in the QoL and 6MWT.

### Implications and Limitations 

The absence of withdrawals and the lack of baseline discrepancies are the study’s strengths. There are some drawbacks to the analysis. The small sample size of this study limits the subgroup analyses of gender and age. Furthermore, measuring the long-term effects of treatment is needed. 

## 5. Conclusions

Both a specialized physical therapy and PBWSTT are effective for children with JMG in terms of enhancing PFTs, neuromuscular functions, and quality of life. Moreover, PBWSTT is an excellent adjunctive to the physical therapy program for those children.

## Figures and Tables

**Table 1:** Patients’ characteristics.

	Group A	Group B	*p*-Value
	Mean ± SD	Mean ± SD	
**Age (years)**	14.20 ± 1.01	14.46 ± 1.06	0.48
**Weight (kg)**	54.73 ± 3.34	55.02 ± 3.02	0.81
**Height (cm)**	157.13 ± 2.82	158.46 ± 2.74	0.20
**BMI (kg/m^2^)**	22.16 ± 1.07	21.89 ± 0.83	0.46
**Sex, n (%)**			
Females	12 (80%)	13 (87%)	0.62
Males	3 (20%)	2 (13%)	
**Severity, n (%)**			
Grade IIA	11 (73%)	12 (80%)	0.66
Grade IIB	4 (27%)	3 (20%)	

BMI, Body mass index; SD, standard deviation; *p*-value, probability value.

**Table 2:** Pulmonary function measures of pre- and post-treatment of both groups.

	Group A	Group B		
	Mean ± SD	Mean ± SD	MD	*p*-Value
**FVC (L)**				
Pre-treatment	1.39 ± 0.08	1.38 ± 0.07	0.01	0.64
Post-treatment	1.54 ± 0.11	1.46 ± 0.07	0.08	0.02 *
MD (% of change)	0.15 (10.8%)	0.08 (5.8%)		
	p = 0.001 *	p = 0.001 *		
**FEV1 (L)**				
Pre-treatment	1.28 ± 0.06	1.29 ± 0.07	-0.01	0.60
Post-treatment	1.41 ± 0.08	1.34 ± 0.05	0.07	0.01 *
MD (% of change)	0.13 (10.16)	0.05 (3.88)		
	p = 0.001 *	p = 0.001 *		
**PEFR** **(L/min)**				
Pre-treatment	166.40 ± 1.84	165.46 ± 1.51	0.94	0.14
Post-treatment	179.53 ± 1.30	171.80 ± 1.08	7.73	0.001 *
MD (% of change)	13.13 (7.89%)	6.34 (3.83%)		
	p = 0.001 *	p = 0.001 *		
**MVV (L/min)**				
Pre-treatment	43.26 ± 1.27	42.66 ± 1.23	0.60	0.20
Post-treatment	49.20 ± 1.32	47.13 ± 1.06	2.07	0.001 *
MD (% of change)	5.94 (13.73%)	4.47 (10.48%)		
	p = 0.001 *	p = 0.001 *		

FVC, forced vital capacity; FEV1, forced expiratory volume in one second; PEFR, peak expiratory flow rate; MVV, maximum voluntary ventilation; SD, standard deviation; MD, mean difference; *p*-value: probability value; * significant at *p* < 0.05.

**Table 3:** CMAP amplitude, isometric muscle force, PedsQL TM MFS, PedsQL, and 6MWD of both groups.

	Group A	Group B		
	Mean ± SD	Mean ± SD	MD	*p*-Value
**CMAP amplitude of rectus femoris** **(mV)**				
Pre-treatment	4.13 ± 0.35	4.20 ± 0.41	−0.07	0.63
Post-treatment	7.33 ± 0.72	5.93 ± 0.61	1.40	0.001 *
MD (% of change)	3.2 (77.48%)	1.73 (41.19%)		
	p = 0.001 *	p = 0.001 *		
**CMAP amplitude of biceps** **(mV)**				
Pre-treatment	5.33 ± 1.11	5.20 ± 0.94	0.13	0.72
Post-treatment	7.46 ± 0.83	6.93 ± 1.09	0.53	0.14
MD (% of change)	2.13 (39.96)	1.73 (33.27)		
	p = 0.001 *	p = 0.001 *		
**Isometric muscle force of biceps brachii (kg)**				
Pre-treatment	12.20 ± 0.86	11.80 ± 0.77	0.40	0.19
Post-treatment	18.73 ± 1.22	18.20 ± 1.08	0.53	0.21
MD (% of change)	6.53 (53.52%)	6.40 (54.24%)		
	p = 0.001 *	p = 0.001 *		
**Isometric muscle force of rectus femoris** **(kg)**				
Pre-treatment	22.93 ± 0.96	22.73 ± 0.79	0.2	0.54
Post-treatment	30. 60 ± 1.05	26.60 ± 1.29	4	0.001 *
MD (% of change)	7.67 (33.45%)	3.87 (17.03%)		
	p = 0.001 *	p = 0.001 *		
**PedsQL TM MFS**				
Pre-treatment	70.86 ± 0.91	70.40 ± 0.73	0.46	0.13
Post-treatment	77.80 ± 0.86	74.33 ± 0.72	3.47	0.001 *
MD (% of change)	6.94 (9.79%)	3.93 (5.58%)		
	p = 0.001 *	p = 0.001 *		
**PedsQL** **total composite**				
Pre-treatment	66.86 ± 0.91	66.53 ± 0.51	0.33	0.23
Post-treatment	77.33 ± 0.48	71.60 ± 0.51	5.73	0.001 *
MD (% of change)	10.47 (15.66%)	5.07 (7.62%)		
	p = 0.001 *	p = 0.001 *		
**6MWD (m)**				
Pre-treatment	311.46 ± 2.35	310.66 ± 2.58	0.80	0.38
Post-treatment	350 ± 2.36	330.73 ± 2.65	19.27	0.001 *
MD (% of change)	38.54 (12.37%)	20.07 (6.46%)		
	p = 0.001 *	p = 0.001 *		

CMAP, compound motor action potential; MD, mean difference; *p*-value, probability value; PedsQL, pediatric Quality of Life; PedsQL TM MFS: pediatric quality of life inventory multidimensional fatigue scale; *p*-value, probability value; 6MWD, six-minute walk distance; SD, standard deviation; * significant at *p* < 0.05.

**Table 4:** Mean stability indices of both groups.

	Group A	Group B		
	Mean ± SD	Mean ± SD	MD	*p*-Value
**APSI**				
Pre-treatment	1.57 ± 0.08	1.54 ± 0.09	0.03	0.31
Post-treatment	1.04 ± 0.05	1.40 ± 0.09	−0.36	0.001 *
MD (% of change)	0.53 (33.76%)	0.14 (9.09%)		
	p = 0.001 *	p = 0.001 *		
**MLSI**				
Pre-treatment	1.80 ± 0.07	1.76 ± 0.09	0.04	0.31
Post-treatment	1.17 ± 0.11	1.56 ± 0.06	−0.39	0.001 *
MD (% of change)	0.63 (35%)	0.20 (11.36%)		
	p = 0.001 *	p = 0.001 *		
**OSI**				
Pre-treatment	1.97 ± 0.09	1.96 ± 0.12	0.01	0.87
Post-treatment	1.37 ± 0.07	1.64 ± 0.09	−0.27	0.001 *
MD (% of change)	0.60 (30.46%)	0.32 (16.33%)		
	p = 0.001 *	p = 0.001 *		

APSI: anterior–posterior stability index; MLSI: medial–lateral stability index; MD, mean difference; OSI: overall stability index; *p*-value, probability value; SD, standard deviation; * significant at *p* < 0.05.

## Data Availability

The data are available in a publicly accessible repository.
